# Supplementary material for: Plasmodium falciparum serology: A comparison of two protein production methods for analysis of antibody responses by protein microarray
Source: PLoS One. 2022 Aug 29;17(8):e0273106. doi: 10.1371/journal.pone.0273106 (PMC9423672; doi:10.1371/journal.pone.0273106)
Supplement: S2 Fig — Correlogram of multiple antigen-matched targets (left). Spearman’s rank correlation reported (rs) and increasing blue colour scale indicates relative strength of correlation based on calculated correlations for all proteins included in this analysis. Protein schematic (right) represents amino-acid aligned representation of IVTT (green) and purified (orange) proteins to the full-length native protein (grey). Proteins in the correlogram and schematic are correspondingly aligned. (PDF) [file pone.0273106.s002.pdf]

S2 Fig. Correlogram of multiple antigen-matched targets (left). Spearman's rank correlation reported (r_s) and increasing blue colour scale indicates relative strength of correlation based on calculated correlations for all proteins included in this analysis. Protein schematic (right) represents amino-acid aligned representation of IVTT (green) and purified (orange) proteins to the full-length native protein (grey). Proteins in the correlogram and schematic are correspondingly aligned.

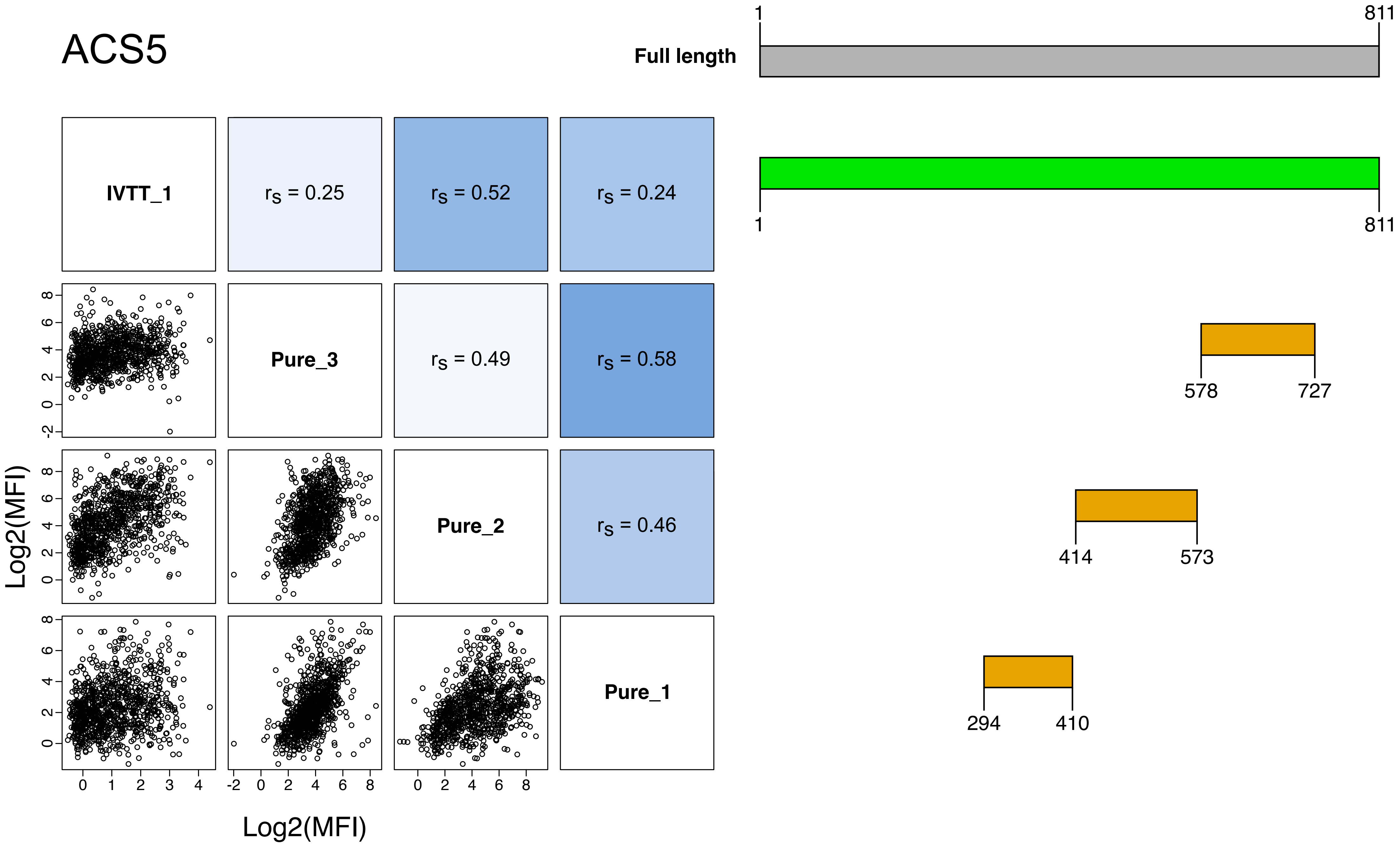

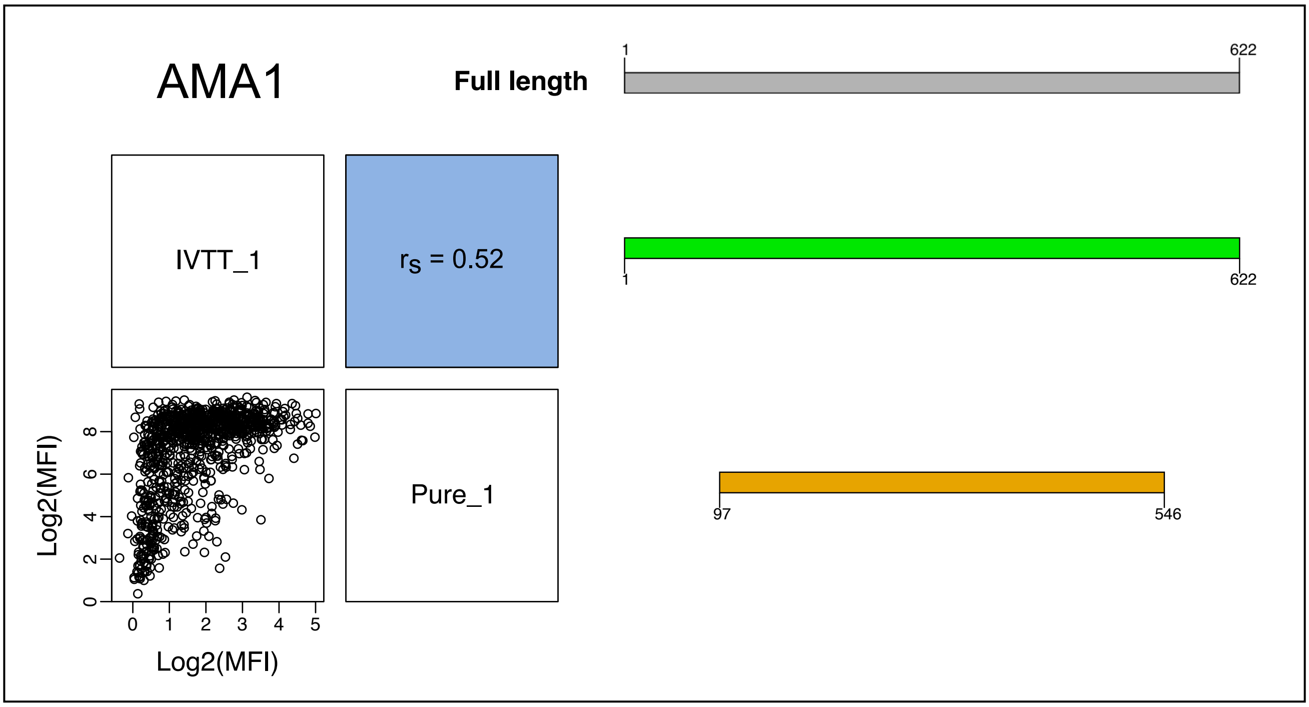
AMA1
Full length
1
622
IVTT_1
rs = 0.52
1
622
Pure_1
97
546
Log2(MFI)
Log2(MFI)

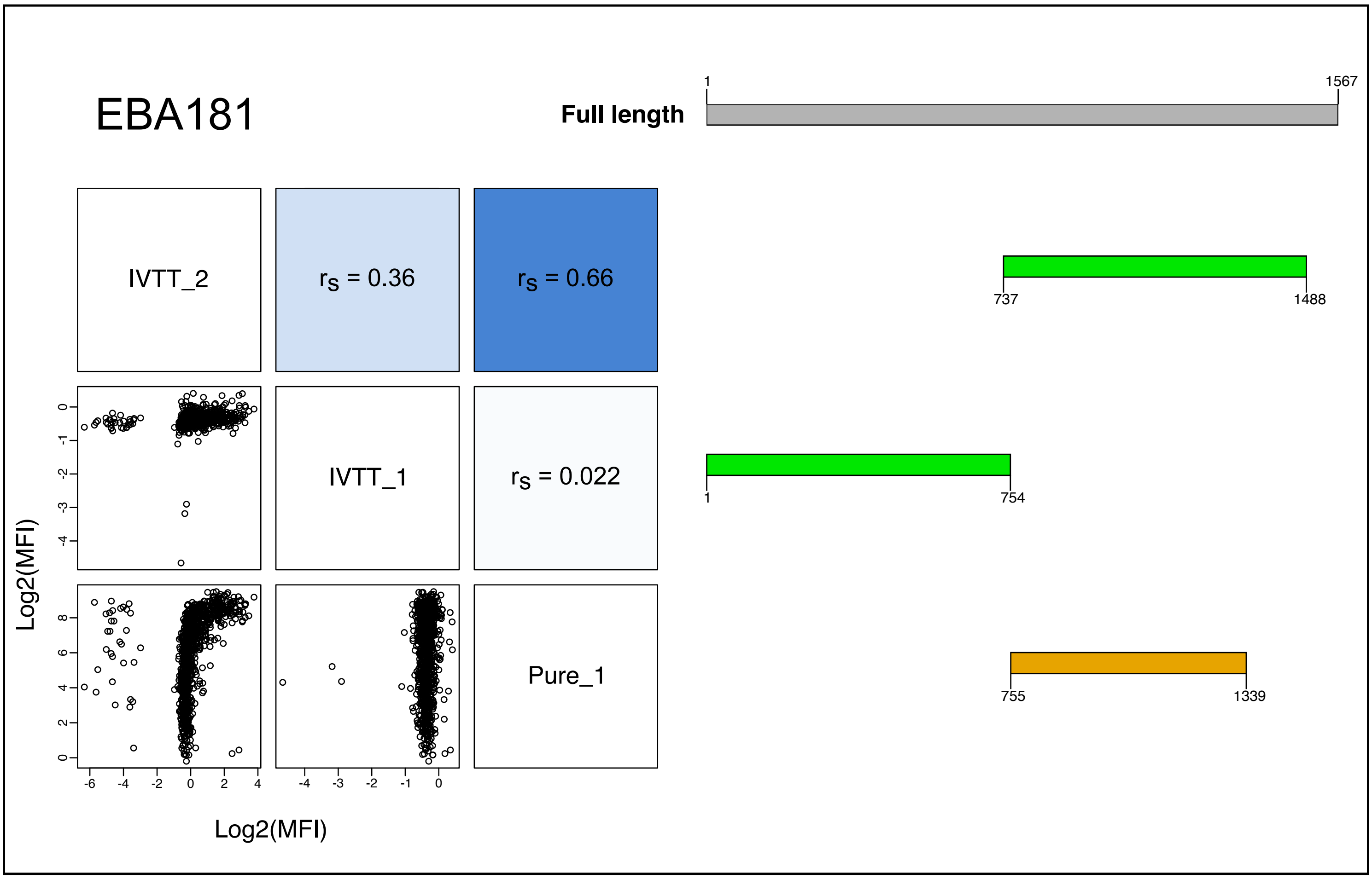
EBA181
Full length
1
1567
IVTT_2
rs = 0.36
rs = 0.66
737
1488
IVTT_1
rs = 0.022
1
754
Pure_1
755
1339
Log2(MFI)
Log2(MFI)

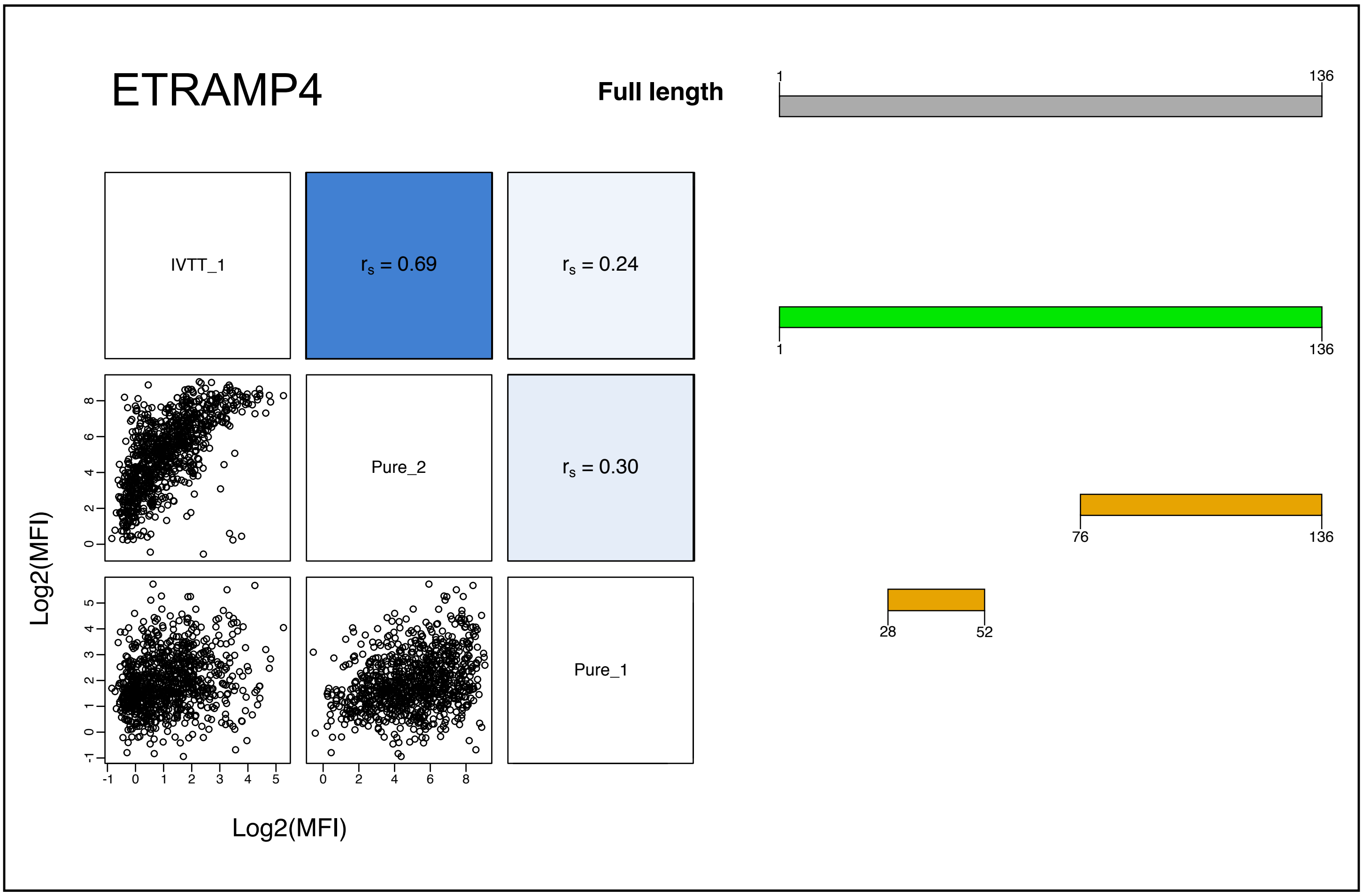
ETRAMP4
Full length
1
136
IVTT_1
r_s = 0.69
r_s = 0.24
1
136
Pure_2
r_s = 0.30
76
136
28
52
Pure_1
Log2(MFI)
Log2(MFI)

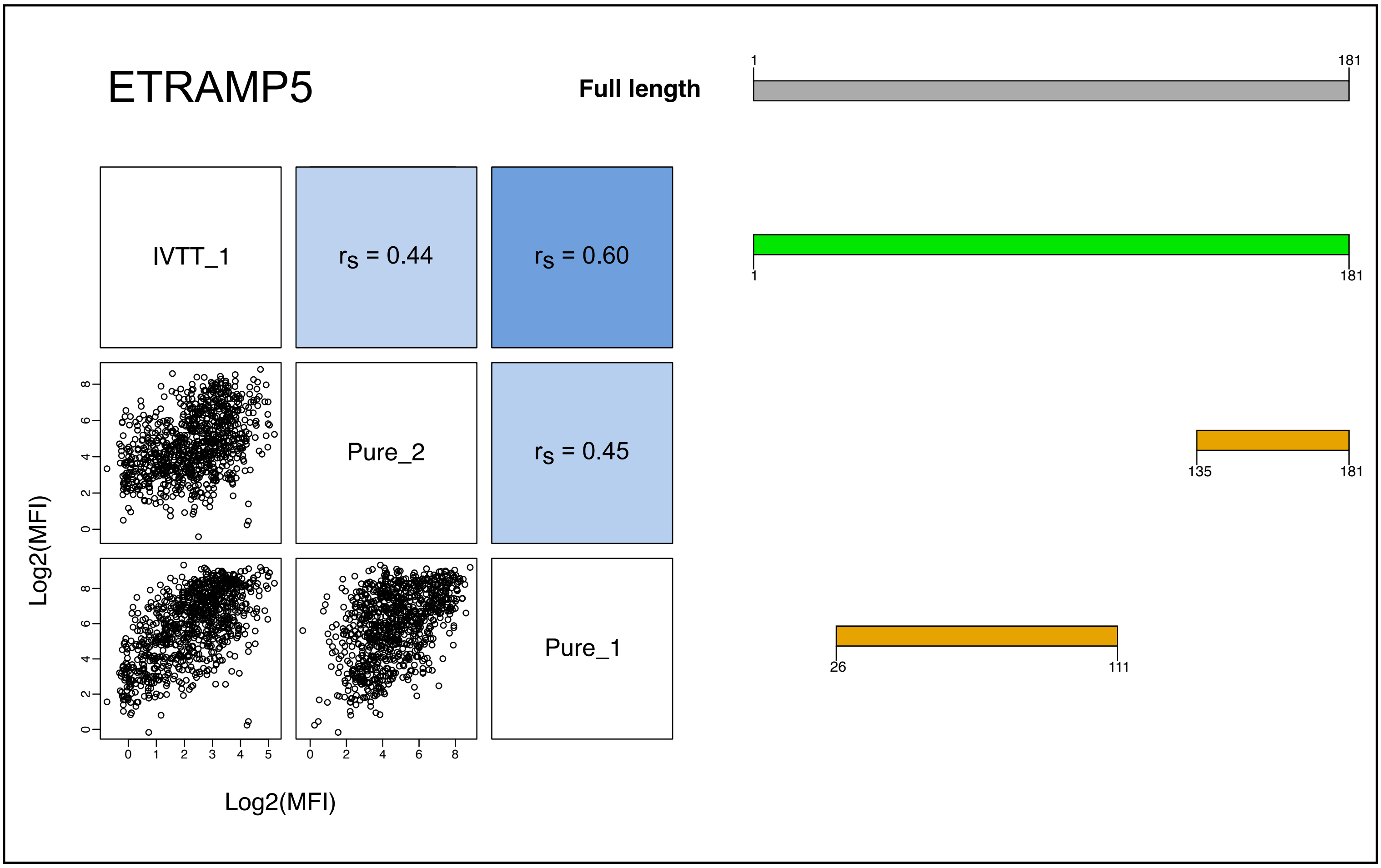
ETRAMP5
Full length
1
181
IVTT_1
r_s = 0.44
r_s = 0.60
1
181
Pure_2
r_s = 0.45
135
181
26
111
Pure_1
Log2(MFI)
Log2(MFI)

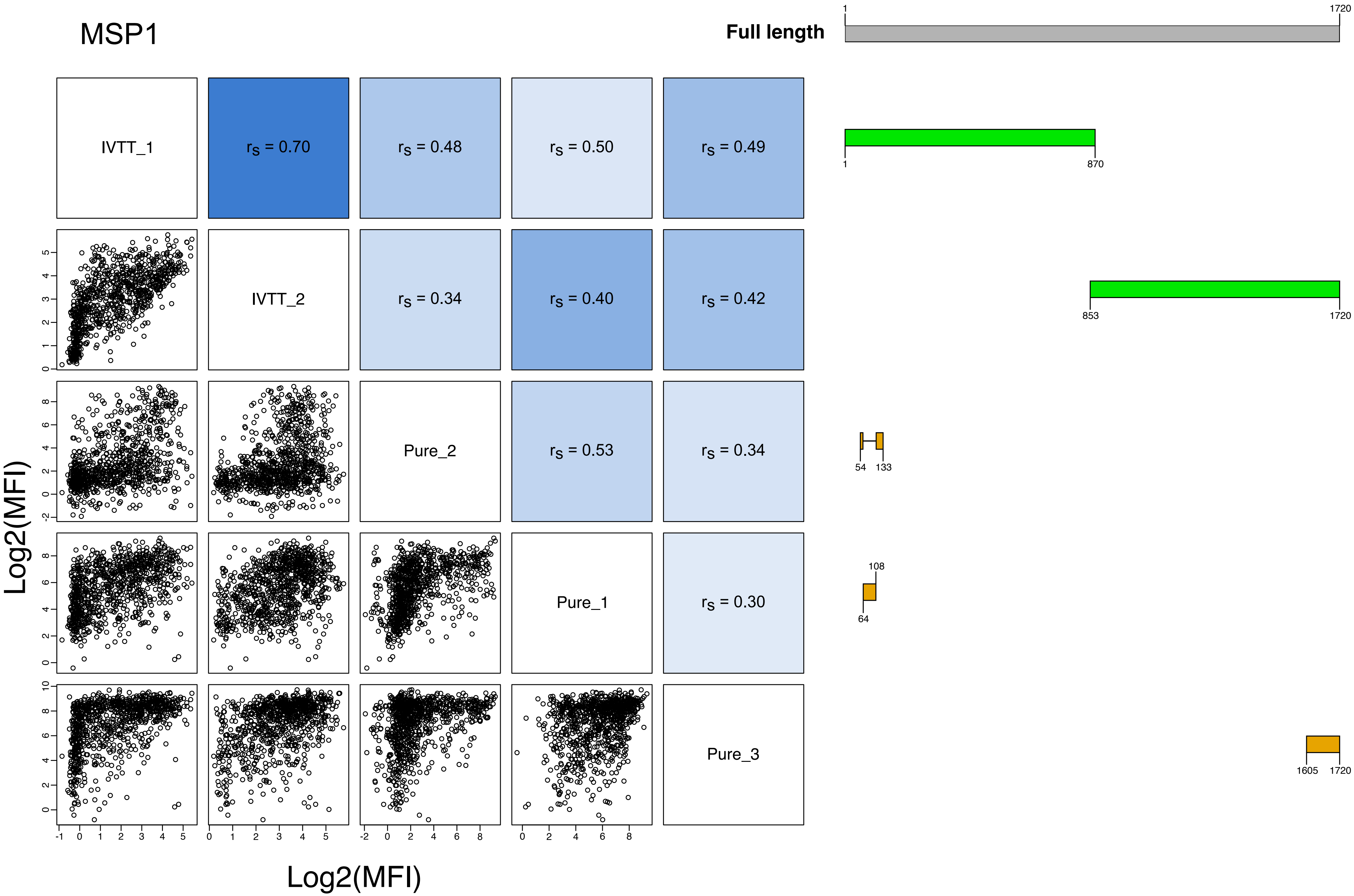

MSP1
Full length
1
1720
IVTT_1
rS = 0.70
rS = 0.48
rS = 0.50
rS = 0.49
870
IVTT_2
rS = 0.34
rS = 0.40
rS = 0.42
853
Pure_2
rS = 0.53
rS = 0.34
54
133
Pure_1
rS = 0.30
108
64
Pure_3
1605
Log2(MFI)
Log2(MFI)

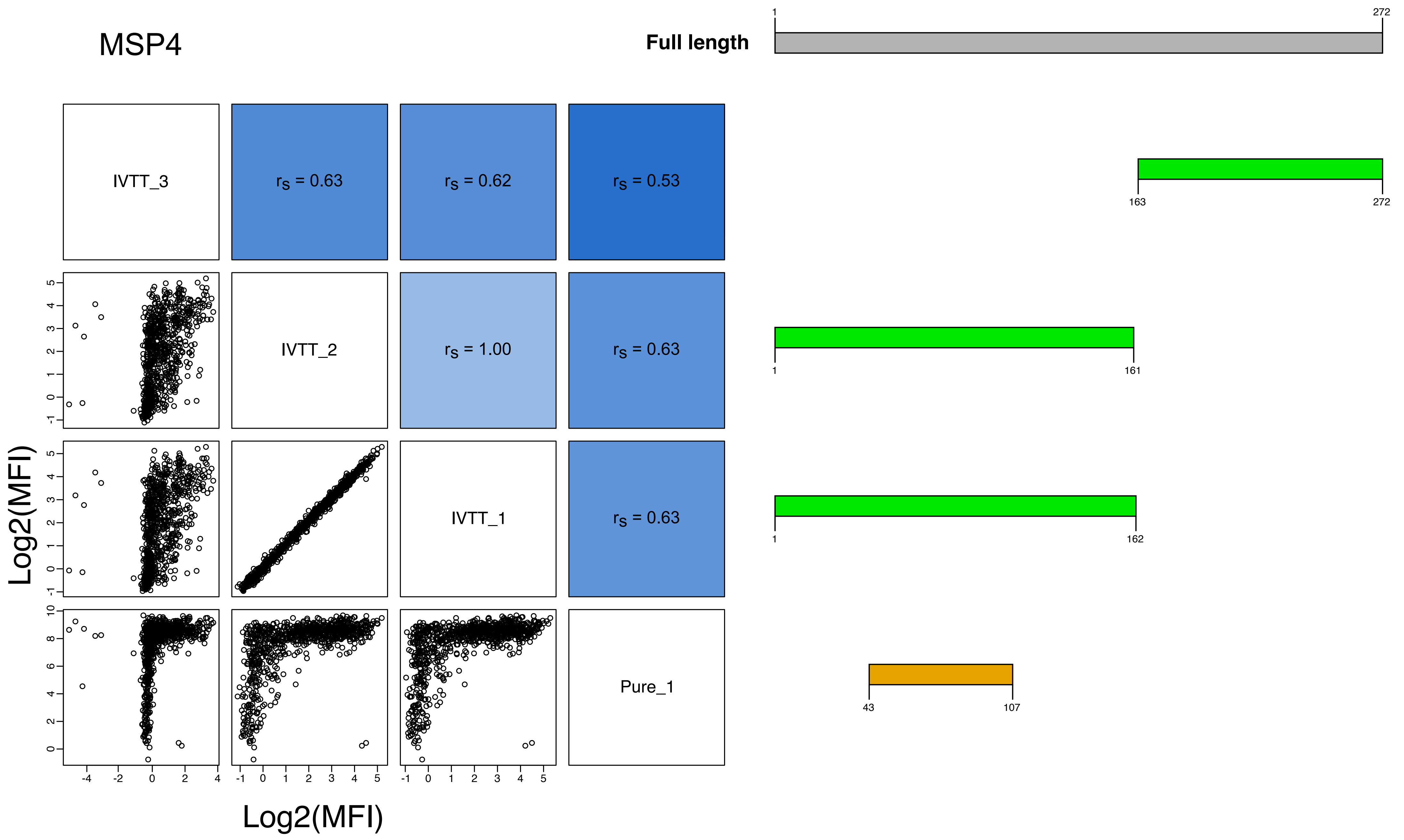

MSP4
Full length
1
272
IVTT_3
rs = 0.63
rs = 0.62
rs = 0.53
163
272
IVTT_2
rs = 1.00
rs = 0.63
1
161
IVTT_1
rs = 0.63
1
162
Pure_1
43
107
Log2(MFI)
Log2(MFI)

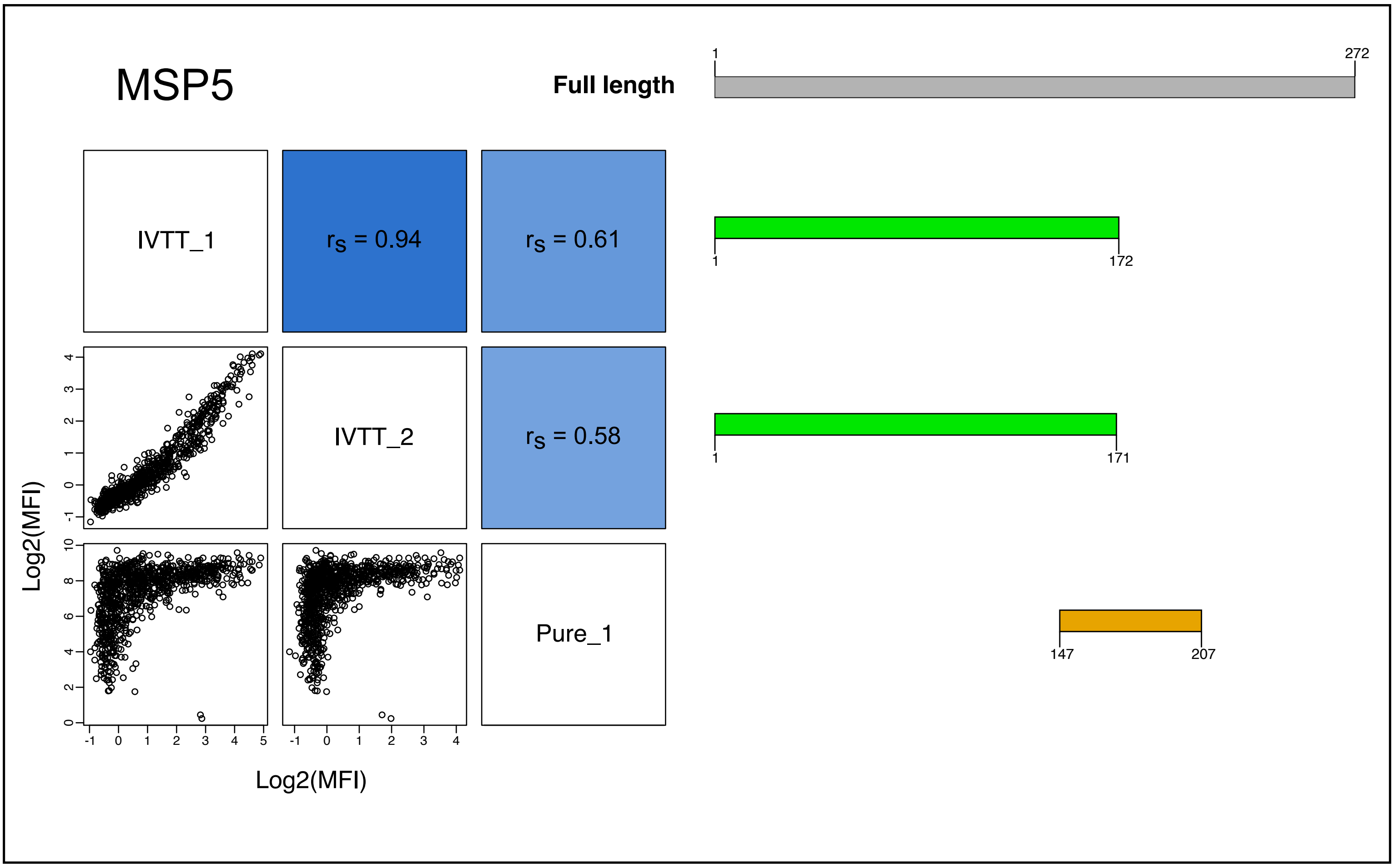
MSP5
Full length
1
272
IVTT_1
rs = 0.94
rs = 0.61
1
172
IVTT_2
rs = 0.58
1
171
Pure_1
147
207
Log2(MFI)
Log2(MFI)

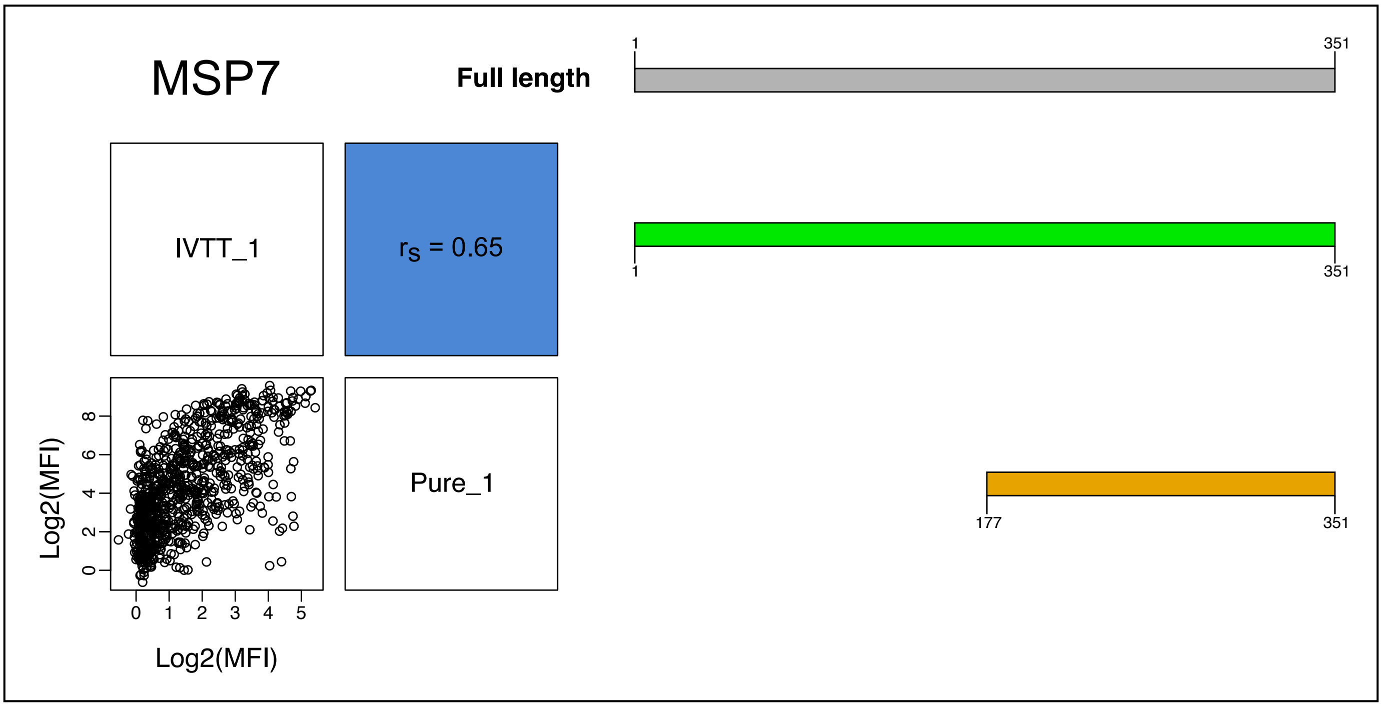
MSP7
Full length
1
351
IVTT_1
rs = 0.65
1
351
Pure_1
177
351
Log2(MFI)
Log2(MFI)

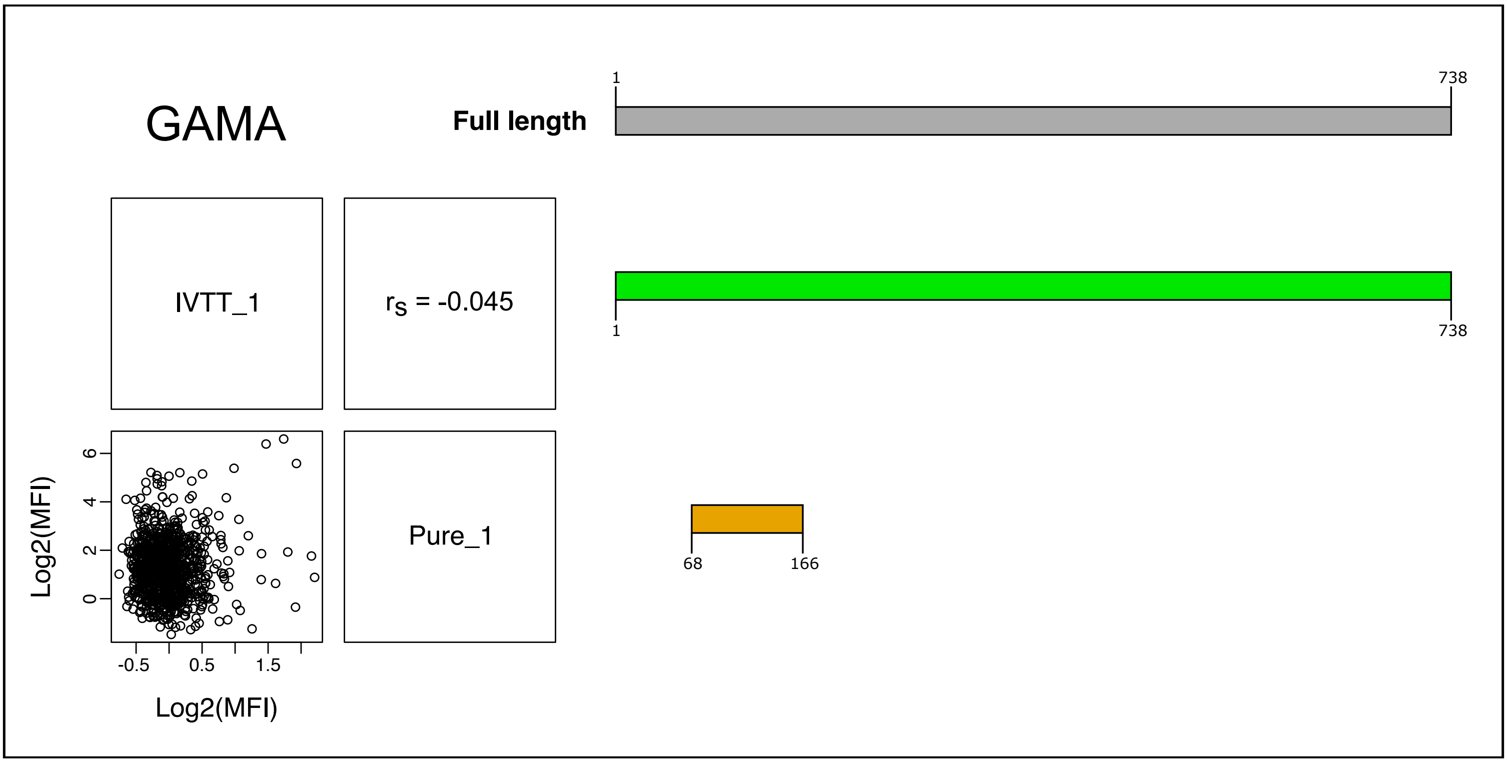
GAMA
Full length
1
738
IVTT_1
r$_S$ = -0.045
1
738
Pure_1
68
166
Log2(MFI)
Log2(MFI)
-0.5
0.5
1.5
0
2
4
6
