# Supplementary material for: Plasmodium falciparum serology: A comparison of two protein production methods for analysis of antibody responses by protein microarray
Source: PLoS One. 2022 Aug 29;17(8):e0273106. doi: 10.1371/journal.pone.0273106 (PMC9423672; doi:10.1371/journal.pone.0273106)
Supplement: S3 Fig — All sample responses (n = 899) to all protein targets grouped by antigen, presented with median and interquartile range. (PDF) [file pone.0273106.s003.pdf]

S3 Fig. Magnitude and range of response to IVTT and purified proteins, stratified by age. All sample responses (n = 899) to all protein targets grouped by antigen, presented with median and interquartile range.

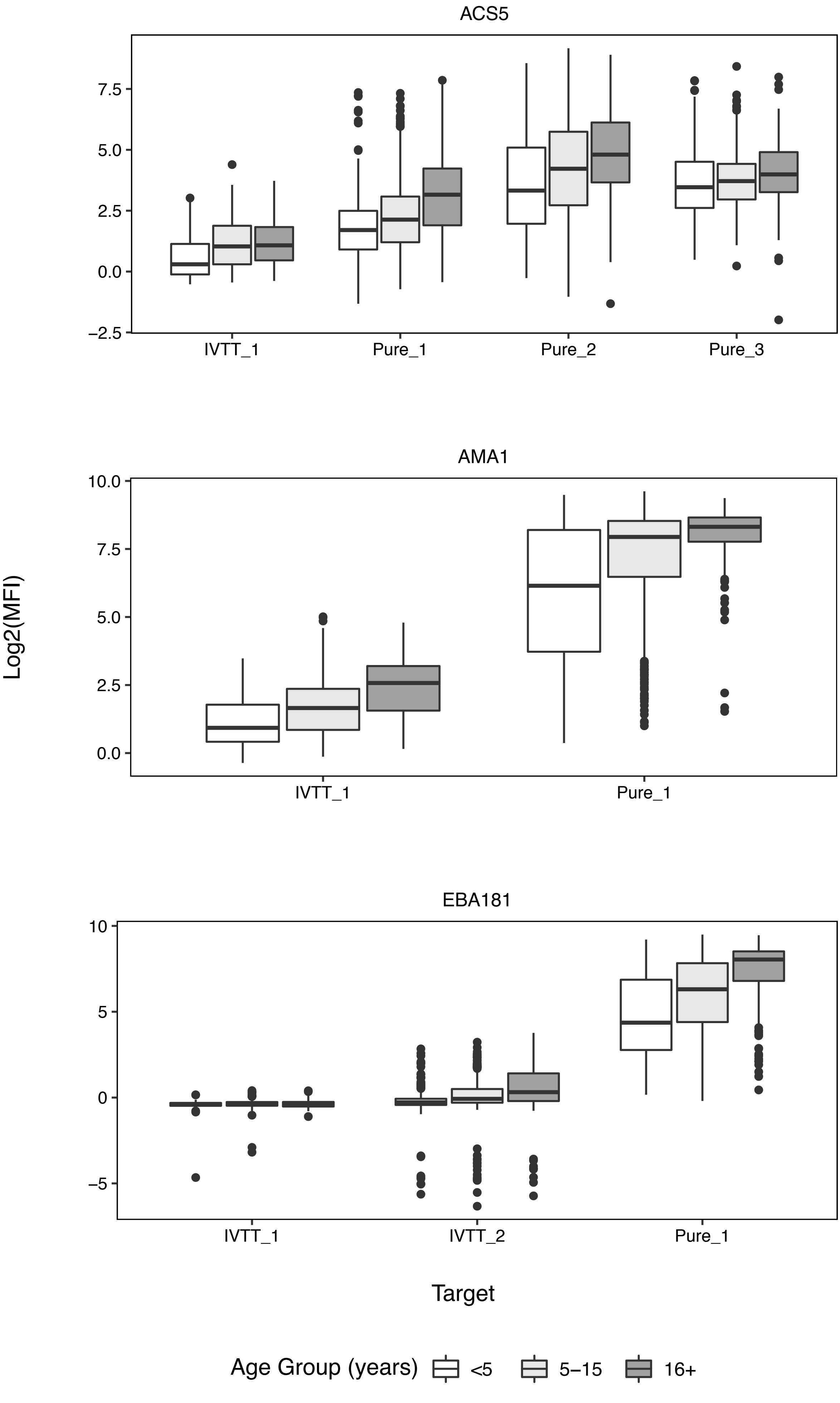

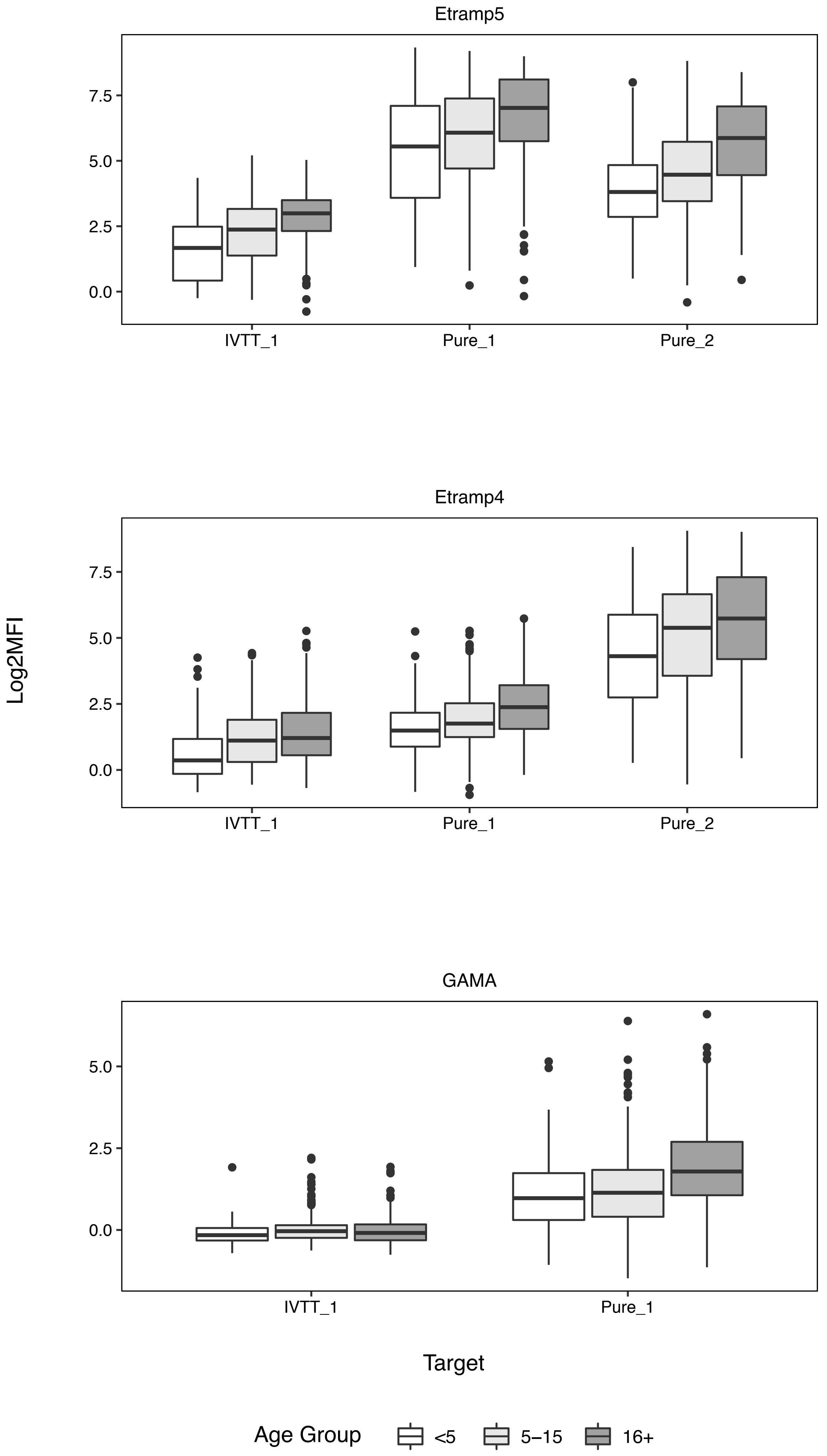
Etramp5
7.5
5.0
2.5
0.0
IVTT_1
Pure_1
Pure_2
Etramp4
7.5
5.0
2.5
0.0
IVTT_1
Pure_1
Pure_2
Log2MFI
GAMA
5.0
2.5
0.0
IVTT_1
Pure_1
Target
Age Group
<5
5−15
16+

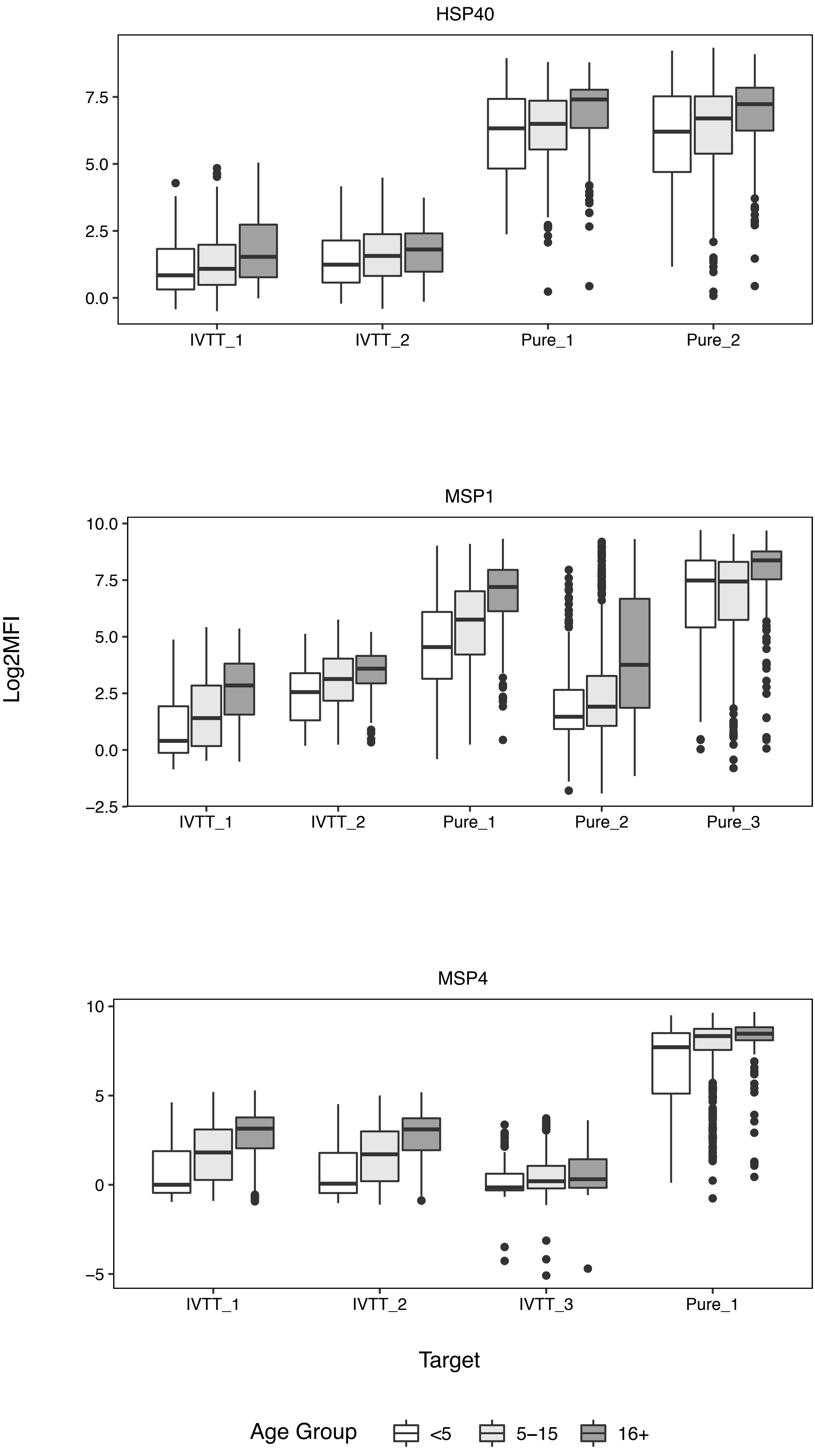

HSP40
7.5
5.0
2.5
0.0
IVTT_1
IVTT_2
Pure_1
Pure_2
MSP1
10.0
7.5
5.0
2.5
0.0
−2.5
IVTT_1
IVTT_2
Pure_1
Pure_2
Pure_3
Log2MFI
MSP4
10
5
0
−5
IVTT_1
IVTT_2
IVTT_3
Pure_1
Target
Age Group
<5
5−15
16+

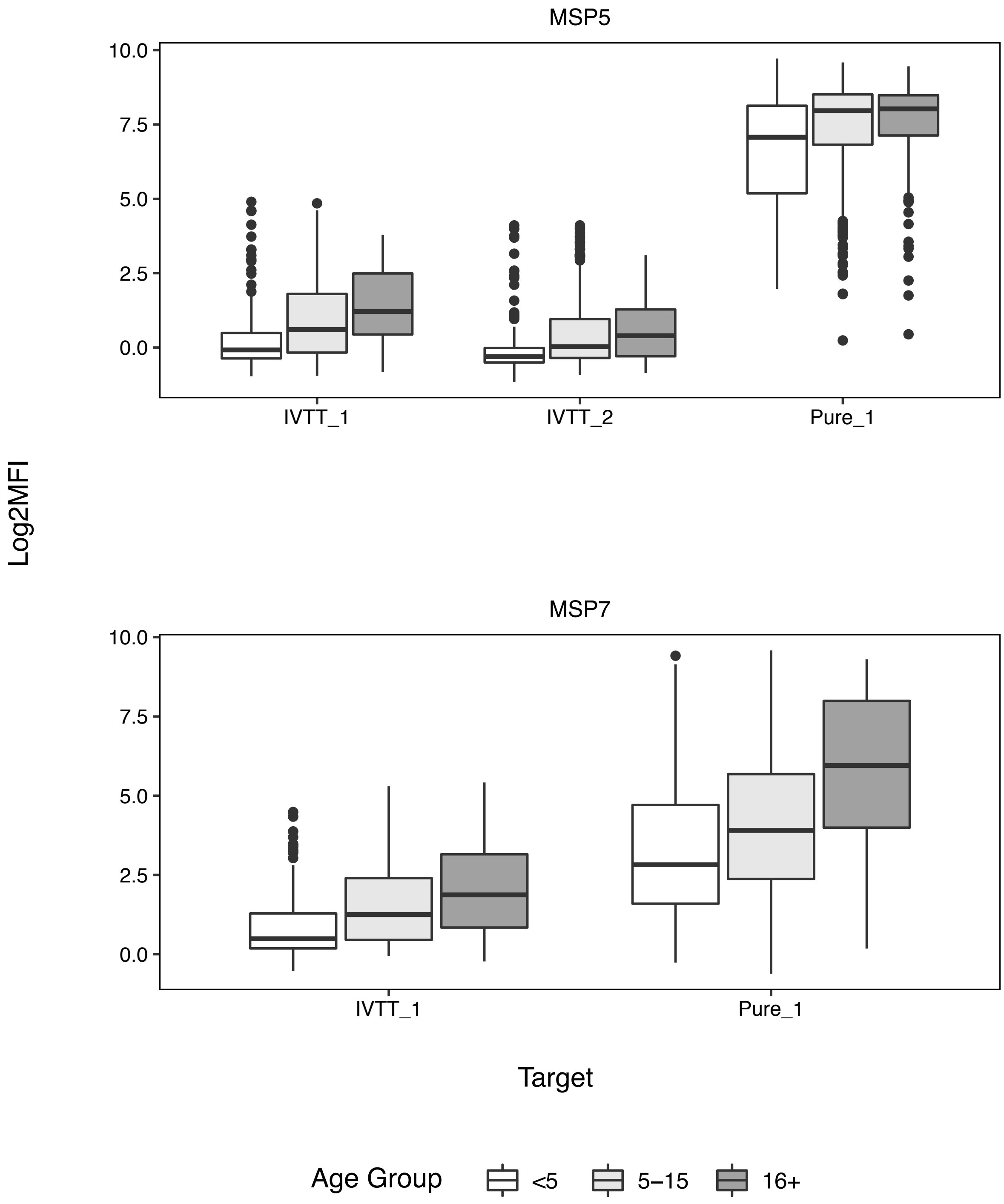
MSP5
10.0
7.5
5.0
2.5
0.0
IVTT_1
IVTT_2
Pure_1
Log2MFI
MSP7
10.0
7.5
5.0
2.5
0.0
IVTT_1
Pure_1
Target
Age Group
<5
5–15
16+
